# Eighth Annual Meeting of the American Society of Dental Surgeons

**Published:** 1847-10

**Authors:** 


### Miscellaneous Notices.

### EIGHTH ANNUAL MEETING OF THE AMERICAN SOCIETY.

The eighth annual meeting of the American Society of Dental Surgeons convened pursuant to adjournment, at Saratoga Springs, Tuesday, August 3d, 1847.

The Society convened at the United States Hotel, at 9 A. M., and adjourned to the Union Hall, where rooms had been provided for holding its daily sessions. At 9½ o'clock the Society met at the latter place and was called to order by the President. Present, E. Parmly, C. A. Harris, A. Westcott, J. Parmly, Lewis Roper, E. Noyes, E. Laroque, Geo. Merriman, C. C. Allen, E. J. Dunning, Benj. Lord, J. H. Foster, J. B. Rich, C. S. Rowell, John Lovejoy, A. Van Camp, Alex. Nelson, H. N. Fenn, Asa Hill, Wm. H. Goddard, J. M. Peak, N. C. Keep, Seth P. Miller, Wm. H. Dwinelle, H. H. Young, E. Baker, D. R. Parmly, H. F. Smith.

The first business was to call upon the Recording Secretary for his report in regard to the duty assigned him relative to the subject of amalgams. This report is as follows, viz.

At the annual meeting of 1846, the American Society of Dental Surgeons made it my duty to issue a protest against the use of amalgams for dental purposes, in accordance with certain resolutions passed at said session, and to direct it to all who were reported as "delinquents" in regard to signing a similar protest ordered by the Society in 1845, and which was then directed to every member of the Society. The number reported as delinquents at the meeting of 1846, or on the original protest, was *fifty six*. I am happy to be able to state that this list is now reduced to *twenty-one*.

The names of said delinquents are as follows—C. C. Allen, E. Baker, Geo. E. Haws, John Lovejoy, E. Laroque, D. Morrow, Geo. Merriman, C. S. Rowell, E. M. Bissel, S. Dillingham, J. Smith Dodge, Nehemiah Dodge, P. Houston, E. Jamet, Benj. Lord, Jas. Taylor, Geo. W. Parmly, Elijah Bryon, N. C. Keep, J. M. Peak and B. B. Brown.

Several of these have replied to the circular by letter, which communications I herewith transmit.

A. WESTCOTT, *Recording Secretary.*

On motion of Wm. H. Goddard, it was unanimously

*Resolved,* That this report, together with the replies elicited by the circular, be referred to a committee of five, and that this committee be requested to report some plan of disposing of this subject at this meeting, and also that said committee be requested to arrange the order of business.

This committee was appointed by the chair, and is as follows, viz.—Drs. C. A. Harris, A. Westcott, W. H. Goddard, J. H. Foster, and J. Parmly.

The committee, after consulting upon this subject, and finding that each member entertained similar views, declined acting till they had consulted with some of those who entertained views upon this subject differing from that expressed by those composing the committee. The committee therefore sent a request to the Society, that at least two more be added, and that they be selected from those who had dissented from the former action of the Society. Whereupon, C. C. Allen and Benj. Lord were added to the original committee. There being no business before the Society, it was resolved to adjourn till 3 o'clock, to give the committee time to deliberate and report upon the subject referred to them.

*Three o'clock, P. M.*

The Society having convened pursuant to adjournment, the committee to whom was referred the Recording Secretary's report presented the following resolutions:

*Resolved,* That your committee will not recommend the expulsion of any member who is not in the practice of *using or recommending* the use of amalgam for filling teeth.

*Resolved further,* That it is the duty of every member, who has the usefulness of this Society at heart, either to acquiesce in its doings so far as his *practice* and *influence* is concerned, or quietly to retire and resign his membership.

C. A. HARRIS,
J. H. FOSTER,
WM. H. GODDARD,
AMOS WESTCOTT, } *Committee.*
J. PARMLY,
BENJ. LORD,
CHAS. C. ALLEN,

On motion of Dr. Merriman, the report was unanimously accepted.

On motion, it was laid on the table for half an hour, that its bearing might be more fully considered by each member; meanwhile the Society proceeded with other business.

On motion of Dr. C. A. Harris, the following resolutions, prepared by Dr. C. O. Cone, were unanimously adopted, viz.

For the purpose of more fully carrying out the design of this Association,

*Resolved,* That the American Society of Dental Surgeons shall, at its annual meetings, appoint a committee of five of its active members, who shall be known as a Committee of Practical Dentistry; the duty of which shall be to make an annual report to the Society of the improvements effected in this country in the management of disease coming within the scope of the dental practitioner, and the condition and progress of dental knowledge in America during the year of their service.

*And be it further Resolved,* That it shall be the duty of each member of this Association to communicate all information or novel results in practice, at their earliest possible convenience, to the above committee, that the above resolution may be more fully carried out.

The following members were appointed on that committee, viz. Drs. C. O. Cone, J. B. Rich, J. H. Foster, E. Maynard, A. Nelson.

On motion of Wm. H. Goddard, the following resolution was adopted:

*Resolved,* That any member in arrears to this Society shall be ineligible to fill any office, and shall be debarred the privilege of voting upon any question pending before the Society, until such arrearages are paid.

The report of the committee was again taken up, and on motion of A. Westcott, it was unanimously adopted.

The Society now proceeded to act upon the cases of individual members composing the list of delinquents.

The first name of this list being that of C. C. Allen, he was called upon to define his position in regard to the subject of amalgams, and also of the action of the Society thereupon. Dr. Allen responded in substance as follows: He stated that two years ago he signed a protest to the effect that he would neither use nor recommend amalgams for dental purposes, that since that time he had not used it, and that he was willing to adhere to the pledge he had already signed. He farther stated, that if it would be acceptable, he would produce that pledge and have it put on file with papers of the Society. He, however, still protested against the action of the Society, on the ground that it was unconstitutional.

Dr. Allen's case was deferred till his pledge was read before the Society.

The name of E. Baker was next called, but he being absent, action on his case was postponed till to-morrow, when it was expected he would be present.

The name of Geo. E. Hawes was next called. Dr. Hawes was not present, but Dr. C. C. Allen answered to his name, and stated that he was authorized to vouch for him. No action was therefore taken upon his case.

Dr. John Lovejoy was next called upon. Dr. Lovejoy responded in substance as follows: That he had used the amalgam, more or less, for filling teeth, and that he had found it beneficial in certain cases, and that he was unwilling to relinquish his "right of opinion;" that in *his* opinion the Society had no right to expel him, or any other member, on the ground assumed by them, and that he should continue to use amalgam as heretofore.

After Dr. Lovejoy had concluded his remarks, A. Westcott offered the following resolution (A,) which was seconded by J. H. Foster, and carried by a two-third vote:

*Resolved,* That Dr. John Lovejoy be and he is hereby expelled from the American Society of Dental Surgeons, for refusing to comply with their positive mandates, in *using*, and in refusing to discontinue the use of amalgams for filling teeth.

Mr. Laroque was next called upon. His views were in substance like those given by Dr. Lovejoy; said he had used the "paste" and was unwilling to abandon it. He was accordingly expelled on the above resolution (A.)

On motion of J. B. Rich, Dr. Lord's case was put over till to-morrow, 10 A. M.

On motion of A. Nelson, seconded by E. J. Dunning, Geo. Merriman was unanimously expelled on resolution (A.)

Adjourned to 8 P. M.

*Eight o'clock, P. M.*

The President in the chair. The case of J. M. Peak was laid over till to-morrow, 10 A. M.

Dr. N. C. Keep was next called upon. He stated that he was desirous to retain his membership, but that he had occasional calls for amalgam fillings, when the patients *would* have it, and that he had sometimes used it where he thought it was for the benefit of the patient; said he would be glad to meet the Society on some conciliating ground, and that if they would give him time, he would prepare a protest such as he would be willing to sign. His case was therefore postponed.

D. Morrow was expelled on the following resolution (B,) by a two-third vote:

*Resolved,* That D. Morrow be and he is hereby expelled from the American Society of Dental Surgeons, for refusing to comply with their positive mandates, by refusing to sign the protest of this body relative to the use of amalgams for filling teeth.

Case of C. S. Rowell laid over to 10 o'clock to-morrow.

G. W. Parmly's case laid over for one year, he being out of the country.

E. Bryan expelled on resolution (A.)

C. A. Harris moved that the cases of those remaining be laid over till next annual meeting. Lost.

Dr. J. H. Foster moved that the cases of E. M. Bissel, P. Houston and James Taylor be laid over one year. Carried.

On motion of A. Hill, it was

*Resolved,* That S. Dillingham, Nehemiah Dodge and E. Jamet be expelled on resolution (B.) Carried by a two-third vote.

Adjourned to meet at 9 A. M. to-morrow.

*Wednesday,* 9 *o'clock, A. M.*

Met pursuant to adjournment. After the President called the meeting to order, the minutes of the meeting up to the present time were read, accepted and approved.

Dr. C. O. Cone (through Dr. C. A. Harris,) offered the following resolution:

*Resolved,* That the Treasurer of the American Society of Dental Surgeons be instructed to forward immediately to all that are indebted to the Society, either for subscription to the American Journal and Library of Dental Science or for annual dues to the Society, their accounts; and if not paid, to enforce payment for the Journal by legal or such other means as he shall deem proper, to secure an early liquidation of their indebtedness for the same.

*Resolved,* That the Treasurer be instructed to forward to each subscriber of the American Journal and Library of Dental Science his subscription, on the issue of the *first number* for the volume forthcoming; and at the same time forward to each member of this Association, whose annual dues remain unpaid, a bill of dues for the current year.

C. C. Allen offered the following preamble and resolutions, which were unanimously adopted:

*Whereas,* one of the objects of this Society is to diffuse a knowledge of correct practice in surgical and mechanical dentistry *among all practising dentists,* and to give to all its members the benefits of all improvements in the science, thereby elevating it to the rank of a liberal profession; therefore,

*Resolved,* That it is derogatory to the dignity of this Society to retain as members any person who has secured, or shall hereafter secure, to himself by "letters patent," any invention or improvement in surgical or mechanical dentistry.

*Resolved,* That no man shall be considered an honorable member of this Society, who has secrets in his profession, or refuses to impart any knowledge or information which he may possess on the subject of surgical or mechanical dentistry, *or any of its collateral branches,* to any other member.

Dr. Baker presented the papers in his hands as chairman of the executive committee.

Several members being now present who were absent yesterday, the Secretary was called upon to recapitulate the preceding action of the Society and the next business in order. This being done, it was declared that action on Dr. Baker's case, which had been laid over that he might be present, was the next business in order.

On Dr. Baker's case being called, the Secretary, A. Westcott, resigned *pro tem.* his seat to E. J. Dunning. The President, E. Parmly, also resigned his chair *pro tem.* to Vice-president Dr. Enoch Noyes.

Dr. C. C. Allen moved that the case of Dr. Baker be indefinitely postponed, seconded by N. C. Keep. Lost.

Dr. Goddard moved that the case of Dr. Baker be *now* called up and acted upon, seconded by J. B. Rich. Carried.

Whereupon the President *pro tem.* requested Dr. Baker to state his views in relation to mineral paste.

He responded to the effect, that the action of the Society in relation to this subject had been entirely unconstitutional. He then expressed his dissatisfaction with the Society for having expelled members, while those were absent who took an interest in the proceedings, and who would have been glad to defend the accused. He then took up the general subject of the use of amalgam, and spoke in its defence.

After Dr. Baker had concluded his remarks, it was moved and seconded that Dr. Baker be expelled on resolution (A.)

Before the vote was taken, Dr. Baker arose and objected to the resolution, on the ground that it implied, or might imply, that he used nothing but mineral paste for plugging teeth.

Dr. Baker was replied to on the part of the Society, in effect, that as he (Dr. B.) had frequently acknowledged that he did use this article in his practice, and as he still declared his intention to use it, the Society would equally accomplish their object should they omit, in the ***resolution,*** to make the charge against which he protested; and that if it was more in accordance with his taste to be expelled under resolution (B,) the Society would not object to gratify him in this particular. It was therefore moved and seconded, that Dr. Baker be expelled on resolution (B.)

Dr. Baker called for the ayes and nays, which being taken, stood, ayes 16, nays 3. Dr. Baker was therefore declared to be ***constitutionally*** expelled under resolution (B.)

On motion, adjourned to meet at 3 o'clock, P. M.

*Wednesday, 3 o'clock, P. M.*

The Society met pursuant to adjournment, the President in the chair.

Dr. C. S. Rowell's name was called, but it was found that he had returned home. His case was disposed of by his being vouched for by Dr. E. Parmly and C. C. Allen.

Dr. N. C. Keep informed the Society that he had prepared a protest which he would be willing to sign, and hoped they would find it satisfactory. The protest referred to reads as follows, viz.

"Whereas, a majority of the members of the American Society of Dental Surgeons feel satisfied that injury is, and has been done to the public, by the use of amalgams for filling teeth, out of deference to their views, and being satisfied myself that it is not free from objection, I wish it understood, that I do not recommend or use amalgam as a substitute for gold. (Signed) N. C. KEEP."

After some discussion in regard to the import of this protest, and after

Dr. Keep gave his own exposition of it, in order to get the sense of the meeting upon it, it was moved, that Dr. Keep's protest be deemed satisfactory. *Unanimously* lost.

Dr. Lord offered his pledge, which was unanimously accepted.

J. M. Peak's case was next called up, but it appearing from the treasurer's books, that Dr. Peak had not paid any dues for four years, his case was dropped on the ground that he was not a member.

On motion, it was resolved, that Dr. C. Starr Brewster's case be acted upon, whereupon, Dr. J. H. Foster offered the following resolution, viz.

*Resolved,* That in view of good and sufficient evidence, to the effect that Dr. C. Starr Brewster has been in the habit of using amalgam for filling teeth, and that it was his intention to continue said practice,

*Therefore, Resolved,* That he be no longer considered an honorary member of the American Society of Dental Surgeons.

J. Smith Dodge's case was then taken up, and J. B. Rich, the chairman of the committee formerly appointed to investigate certain charges, reported; that he had summoned Dr. Dodge, and that he had not paid any attention to it: whereupon, he was expelled for *contumacy.*

Dr. C. C. Allen moved that Dr. Solyman Brown's resignation be accepted, on the ground that he had gone out of the profession. This motion was seconded by A. Westcott, and unanimously carried.

On motion of A. Westcott, Dr. Brown's dues were remitted.

On motion of E. Parmly, seconded by A. Westcott, a vote of thanks was tendered to Dr. Brown for his former labors in the society.

The committee on "Tabular Sheet," presented a report, which was accepted, but it not being finished, was referred back to the committee for completion, and publication.

Adjourned to 8 P. M.

*Eight o'clock, P. M.*

The first business of the Society was to elect officers for the ensuing year. The result was as follows:

| | |
|---|---|
| E. PARMLY, New York, | PRESIDENT. |
| LEWIS ROPER, Philadelphia, | 1st VICE-PRESIDENT. |
| E. NOYES, Baltimore, | 2d " " |
| WM. H. GODDARD, Louisville, | 3d " " |
| J. B. RICH, New York, | COR. SECRETARY. |
| A. WESTCOTT, Syracuse, N. Y. | REC. " |
| J. H. FOSTER, New York, | LIBRARIAN. |
| C. A. HARRIS, Baltimore, | 1st EDITOR. |
| A. WESTCOTT, Syracuse, N. Y. | 2d " |
| WM. H. DWINELLE, Cazenovia, N. Y. | 3d " |

*Executive and Examining Committee.*—Drs. J. H. Foster, J. B. Rich, E. J. Dunning, E. Parmly, J. M'Ilhany, C. A. Harris, H. N. Fenn.

*Publishing Committee.*—Editors.

*Opening Address.*—E. Maynard.

*Dissertations.*—1st. J. B. Rich. 2d. H. H. Young. 3d. Asa Hill. 4th. C. C. Allen. 5th. Dr. Miller.

Dr. N. C. Keep tendered his resignation, which was accepted.

The following resolution was offered by J. B. Rich,

*Resolved,* That persons delivering addresses or essays before this Society, be requested to furnish copies of the same, to be placed in the archives of this Society. And it is further

*Resolved,* That the editors of the official Journal of this Society have free access to such addresses and essays as may be deposited in the archives, for the purpose of making such selections for publication as they may see fit.

Dr. B. B. Brown's case was next called up. After due consideration it was, on motion of J. B. Rich,

*Resolved,* That he be expelled on resolution B.

Adjourned till 9 to-morrow.

*Thursday,* 9 *o'clock, A. M.*

On motion of Wm. H. Goddard,

*Resolved,* That each member of this Society be furnished with a list of its officers and members, printed upon a card suitable for framing, and signed, officially, by the President and Recording Secretary, and the seal of society affixed, and that it be published in five different papers, at the discretion of the editors, at the expense of the society.

On motion of A. Westcott, Dr. C. C. Allen was called upon to read the pledge which he had hitherto signed, and which he had requested the society to accept.

Dr. Allen accordingly read it before the Society, but it was decided that it did not embrace the ground covered by the pledge signed by other members, and, further, it did not carry out the spirit of the resolutions unanimously passed by the committee to whom the list of delinquents was referred, and of which committee Dr. Allen was a member. He was therefore requested to draft one in accordance with said resolution, and time was given him to do so.

On motion of Dr. C. C. Allen,

*Resolved,* That the Treasurer be authorised, at the expense of the Society, to take legal counsel in regard to the power of the society to collect annual dues.

The Executive and Examining Committee reported the following named gentlemen as applicants for membership, as being, in their estimation, eligible to the honor, viz. Wm. Dalrymple, C. H. Bestor, Wilks Allen, and Thomas Palmer.

All of the above were unanimously elected. Wm. Dalrymple and Thomas Palmer signed (by proxy) the constitution, and they were, therefore, admitted to full membership.

On motion of Dr. C. C. Allen, it was

*Resolved,* That when the Society adjourn, it adjourn to meet on the first Tuesday of August, 1848, at Niagara Falls.

Dr. C. C. Allen now presented a substitute for the pledge he had offered to the Society. On motion of Dr. Wm. H. Goddard, it was *accepted.*

The committee on Dr. John Allen's case, presented the following preamble and resolutions, viz.

Your committee appointed to take into consideration the case of Dr. John Allen of Cincinnati, relating to the course pursued by him towards this Society in respect to an invention which he has had patented, for remedying deformities of the face, report:

That, whereas, after having freely offered the benefits of his invention to every member of the Society, to be used gratuitously; that without their knowledge he had secured a patent therefor; and that, whereas, he he saw fit, at a subsequent period, to make an attempt to exact from members who might be disposed to use it, a certain per centage for the privilege of using it, your committee deem his conduct highly reprehensible.

And, whereas, Dr. Allen has made such use of the fact of a gold medal having been presented to him by this society, as the society do not contemplate in making such awards, and cannot approve or sanction,

*Therefore, Resolved,* That the censure of the Society be passed upon Dr. Allen, for having acted in a manner derogatory to his character as a professional gentleman, and attended with consequences highly detrimental to the Society of which he is a member.

JNO. B. RICH,
J. H. FOSTER,
J. PARMLY.

The report was accepted, and the committee discharged. On motion of J. B. Rich, the report was unanimously adopted.

On motion of Dr. Dunning,

*Resolved,* That the Corresponding Secretary address communications to the honorary members of this Society, requesting them to inform the Society definitely, what are their views respecting the employment of amalgam for stopping teeth, and if now using it, their intentions with regard to its future use in their practice.

Adjourned to meet at 4 P. M.

*Thursday, 4 P. M.*

Dr. N. C. Keep presented his resignation. Accepted.

An essay was read by Wm. H. Elliott, and, by unanimous vote, it was requested for the archives of the Society.

On motion of Dr. Harris, the vote to meet at Niagara Falls was reconsidered, and finally rescinded. On motion,

*Resolved,* To hold their next meeting at *Saratoga Springs*

On motion, it was

*Resolved,* That the aphorisms be placed in the hands of the *Editors.*

On motion of Dr. C. A. Harris, the Executive Committee were requested to make *the first day's business* of the Society at next annual meeting, *the discussion of the subject of plugging teeth.*

On motion of Dr. Westcott, a vote of thanks was tendered to the proprietors of the Union Hall, for their attention and kindness to the Society while in session.

Adjourned to meet at Saratoga Springs, on the first Tuesday in August, 1848.

